# Recent Progress of Surface-Enhanced Raman Spectroscopy for Bacteria Detection

**DOI:** 10.3390/bios13030350

**Published:** 2023-03-06

**Authors:** Lulu Liu, Wenrui Ma, Xiang Wang, Shunbo Li

**Affiliations:** 1 College of Chemistry and Chemical Engineering, Chongqing University of Science and Technology, Chongqing 401331, China; 2 Key Laboratory of Optoelectronic Technology and Systems, Ministry of Education, College of Optoelectronic Engineering, Chongqing University, Chongqing 400044, China; 3 Key Disciplines Laboratory of Novel Micro-Nano Devices and System Technology, College of Optoelectronic Engineering, Chongqing University, Chongqing 400044, China; 4 Department of Mechanical Engineering, Dongguan University of Technology, Dongguan 523808, China

**Keywords:** bacterial detection, surface-enhanced Raman spectroscopy, microfluidic SERS chip, antimicrobial susceptibility testing

## Abstract

There are various pathogenic bacteria in the surrounding living environment, which not only pose a great threat to human health but also bring huge losses to economic development. Conventional methods for bacteria detection are usually time-consuming, complicated and labor-intensive, and cannot meet the growing demands for on-site and rapid analyses. Sensitive, rapid and effective methods for pathogenic bacteria detection are necessary for environmental monitoring, food safety and infectious bacteria diagnosis. Recently, benefiting from its advantages of rapidity and high sensitivity, surface-enhanced Raman spectroscopy (SERS) has attracted significant attention in the field of bacteria detection and identification as well as drug susceptibility testing. Here, we comprehensively reviewed the latest advances in SERS technology in the field of bacteria analysis. Firstly, the mechanism of SERS detection and the fabrication of the SERS substrate were briefly introduced. Secondly, the label-free SERS applied for the identification of bacteria species was summarized in detail. Thirdly, various SERS tags for the high-sensitivity detection of bacteria were also discussed. Moreover, we emphasized the application prospects of microfluidic SERS chips in antimicrobial susceptibility testing (AST). In the end, we gave an outlook on the future development and trends of SERS in point-of-care diagnoses of bacterial infections.

## 1. Introduction

In the surrounding environment, a variety of pathogenic bacteria such as *Escherichia coli*, *Pseudomonas aeruginosa* and *Staphylococcus aureus* can be found [1]. These pathogens lead to food pollution [2], environmental pollution [3] and wound infection [4], which pose a huge threat to human life. Owing to their high morbidity and mortality rates, pathogenic bacterial infections are a considerable threat to global health [5]. The rapid detection and reliable identification of pathogens have become the focus in many fields, such as food safety [6], public health [7], biological analysis [8], disease diagnosis [9,10] and environmental monitoring [11]. In recent years, the problem of drug resistance has become increasingly serious because of the irrational use of antibiotics [12]. It is essential to identify resistant bacterial strains and evaluate the effects of antibiotics. Traditional detection methods, such as the plate counting method [13], light microscopic examination, enzyme-linked immunosorbent assay (ELISA) [14], polymerase chain reaction (PCR) [15] and mass spectrometry [16], are commonly used for the detection and identification of pathogenic bacteria. However, these well-established methods are either time-consuming, laborious or rely on large equipment and well-trained personnel. For example, the operation steps of colony counting on plates are simple, but test results are usually delayed due to the long time needed for the cultivation of bacteria [17]. PCR for bacteria detection shows high detection sensitivity and specificity, but the operation process is complicated and professional operation skills are also required. ELISA has good specificity, high sensitivity and a fast detection time, but the expensive antibodies and requirement of strict reagent storage always limit its application. The shortcomings of the above-mentioned detection methods of pathogenic bacteria hinder their wide applications in rapid clinical diagnostics, environmental monitoring and food contamination. The development of rapid, simple and highly sensitive detection methods for the qualitative and quantitative detection of pathogenic bacteria has become an urgent demand.

Raman spectroscopy is an analytical technique discovered by the Indian scientist C.V. Raman [18]. It can be applied to analyze the molecular structure based on the molecular vibration and rotation information of scattering spectra [19]. However, it is difficult to detect low-concentration samples via conventional Raman spectroscopy due to the very small cross-section area of Raman scattering [20]. The application field of Raman spectroscopy was greatly limited due to its low intensity and sensitivity [21]. Benefiting from the recent advancements in material science, nanotechnology and optical technology, surface-enhanced Raman scattering (SERS) was developed and widely used in bioanalysis, clinical diagnosis and biomedical research [22,23,24]. In SERS, Raman signals of molecules can be enhanced by six to ten orders of magnitude, owing to the electromagnetic field enhancement and chemical enhancement effects generated by nanostructures [25]. The detection limit of SERS is even as low as the single-molecule level, so SERS is regarded as an ultrasensitive technique [26]. Moreover, SERS is not only free from the interference of water [27] but also causes no damage to test samples [28,29], making it very suitable for analyzing biological samples [30], such as bacteria [31], viruses [32,33] and biomarkers in blood or body fluids [34]. SERS applied for bacteria detection has great advantages, such as a fast detection speed and high sensitivity without using labeling agents such as antibodies, and, in particular, it has simultaneous detection capabilities for multiple types of bacteria [35,36]. In addition, SERS is considered a promising tool to detect bacteria in infected wounds or blood tissue without damaging the actual sample structure [37,38].

In recent years, SERS has blossomed into a rapidly growing research area for the detection of various kinds of bacteria [36,39,40]. Bacteria detection based on SERS can be mainly divided into two categories: the label-free method [34] and the label-based method using SERS tags [41]. Microfluidic technology has the ability to integrate multiple functional modules, such as the separation, enrichment, mixing, reaction and detection of target samples, into one chip, which has the advantages of a lower sample demand, a short reaction time, fast detection, large integration and good portability [42,43,44,45]. The development of a lab-on-a-chip system allows for the successful combination of SERS with microfluidic chips [46]. Once the SERS detection module is integrated into the microfluidic chip, some problems, such as a complicated sample preparation process, poor signal reproducibility and sample contamination in open systems, can be effectively solved [47]. By combining effective SERS substrates and well-designed microchannels, microfluidic SERS chips not only carry out pretreatment operations, such as bacteria sorting, separation and enrichment, but also achieve the effective and convenient detection and identification of pathogenic bacteria [48,49].

In this review, the application of SERS in the fields of pathogenic bacteria detection as well as antibiotic resistance diagnosis was comprehensively discussed. Firstly, the mechanisms of SERS detection and the SERS-active substrate were introduced to help readers understand how the SERS spectra were discovered and generated. Then, we focused on the application of SERS in the ultrasensitive qualitative analysis and quantitative detection of bacteria. Furthermore, we highlighted the recent advancements in microfluidic SERS chips for the enrichment and detection of pathogenic bacteria. Antibiotic resistance has become one of the major global concerns to public health with the excessive and inadequate usage of antibiotics. The microfluidic SERS chip applied for AST was also introduced. This review was concluded with a discussion on the challenges and prospects of SERS-based miniaturized chips or microdevices for the point-of-care diagnosis of bacterial infections.

## 2. Mechanism of SERS

**Figure 1:**
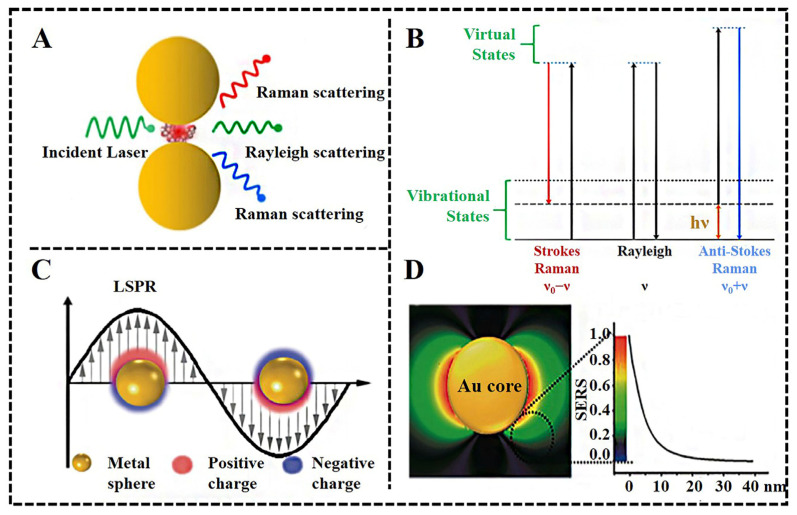
(**A**) Scheme of Raman and Rayleigh scattering of light by a molecule located between two metallic nanoparticles [52]. Reproduced with permission from American Chemical Society Copyright (2022); (**B**) Jablonski diagram representing the quantum energy [52]. Reproduced with permission from American Chemical Society Copyright (2022); (**C**) Schematics of localized surface plasmon resonance (LSPR) [56]. Reproduced with permission from American Chemical Society Copyright (2022); (**D**) Finite difference time domain simulation of the electromagnetic field distribution and dependence of SERS enhancement on the distance from the nanoparticle surface [56]. Reproduced with permission from American Chemical Society Copyright (2022).

In 1928, C.V. Raman found that the scattering frequency of light changed when light passed through a transparent medium and interacted with molecules, now called Raman scattering [50]. As shown in Figure 1A, the incident photons interact with molecules and subsequently generate emitting photons under laser irradiation. In the process, most photons are elastically scattered without exchanging energy (Rayleigh scattering) [51], while a small proportion of photons gain or lose energy, thus leading to a change in both the frequency and direction of the photon (Raman scattering). The differences in energy caused by the vibration and rotation states of the molecules are shown in Figure 1B [52]. The intensity of Rayleigh scattering is only 10^−3^ of the incident light intensity, and the intensity of Raman scattering is only about 10^−3^ of the intensity of Rayleigh scattering, which is 10^−6^ of the incident light intensity. As can be seen, Raman scattering is weak, so some effective strategies need to be used to enhance the Raman signal for the detection of the molecules.

In 1974, Fleischmann and co-workers first reported that the Raman signal of pyridine when in close contact to a rough silver electrode was considerably enhanced [1]. Subsequently, Jeanmaire and Van Duyne discovered that the Raman signal of adsorbed molecules on the surface of a roughened novel metal was significantly enhanced [2]. Since then, the concept of surface-enhanced Raman scattering (SERS) has been proposed and attracted lots of attention. So far, the SERS enhancement theory has still been a controversial matter and has not been clearly explained. It is generally accepted that electromagnetic enhancement and chemical enhancement make major contributions to SERS enhancements [25,53].

Electromagnetic enhancement is the main contributor to the SERS effect and relies on the resonance between the electrons on the surface of a metallic nanostructure and the incident light, namely, the localized surface plasmon resonances (LSPR) [54]. It has been proven that the enhancement factor for electromagnetic fields is approximately proportional to the fourth power of the local electric field intensity generated by metal nanostructures. More importantly, the electromagnetic field around the nanostructures is not uniformly distributed but highly localized in a narrow space called “hot spots” [55]. SERS enhancement declined nearly exponentially with the distance between the interested point and nanostructures (Figure 1D), so only the Raman signal of molecules adsorbed on or very near the surface of the nanostructures can be enhanced [56]. “Hot spots” usually occur in the gaps or sharp vertices of the nanostructures made by noble metals, semiconductors or metal–organic frameworks. Compared with traditional Raman, the SERS signal of molecules near the “hot spots” can be greatly enhanced with an enhancement factor of 10^6^~10^8^, and the density of a “hot spot” is proportional to the enhancement effect [57].

Chemical enhancement is attributed to the electronic-transfer processes between the metallic surface and the adsorbed molecules [58]. The distance of the electronic-transfer effect is limited to within 10 nanometers. Once the incident light is matched with the electron transfer energy of the adsorbed molecules, resonance Raman enhancement can be achieved. This effect brings about a change in the molecular polarization, and the Raman signal of the analyte can be enhanced by two to three orders of magnitude [59]. Compared to electromagnetic enhancement, chemical enhancement makes a lower contribution to the total enhancement of a Raman scattering signal. Unlike electromagnetic enhancement, chemical enhancement is closely related to the chemical structures of the molecules. The correlation between the molecular structures of different organothiols and their SERS enhancement factors can be estimated using a simple internal reference method by Ansar et al. [60].

## 3. SERS Substrates for Bacteria Detection

The SERS enhancement is dependent on the interaction between analyte molecules and the nanostructured surface of substrates. For bacteria analysis via SERS, excellent active SERS substrates are essential to achieve highly sensitive detection of target bacteria as well as ensure good reproducibility of SERS detection [57]. The rapid development of nano-fabrication techniques provides a broad space for the development of SERS-active substrates. An ideal SERS substrate should possess common features, such as high stability, good reproducibility, strong Raman signal enhancement and easy fabrication.

**Figure 2:**
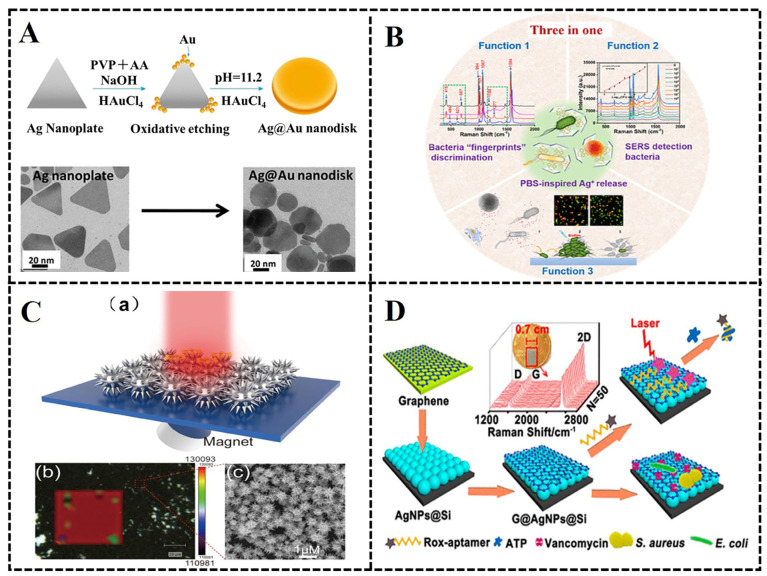
(**A**) The preparation method for Ag@Au nanodisks as SERS substrate [76]. Reproduced with permission from American Chemical Society Copyright (2022); (**B**) SERS substrate made of smart, triple-functional Au-Ag-stuffed nanopancakes for discrimination, sensitive detection and inactivation of different pathogenic bacteria [77]. Reproduced with permission from American Chemical Society Copyright (2022); (**C**) SERS substrate made of magnetic-based, flower-like silver composite microspheres (Fe_3_O_4_@SiO_2_@Ag microflowers). (a) Schematic of the in-situ SERS detection of R6G, (b) Area mapping of the SERS signal of R6G, (c) SEM image of the Fe_3_O_4_@SiO_2_@Ag microflowers [78]. Reproduced with permission from Royal Society of Chemistry Copyright (2022); (**D**) Graphene (G)−silver nanoparticle (AgNP)-silicon (Si) sandwich nanohybrids (G@AgNPs@Si) for SERS sensing applications ranging from the molecular to cellular [80]. Reproduced with permission from American Chemical Society Copyright (2022).

Generally, SERS substrates can be divided into nanocolloids and solid-based nanostructure substrates [61,62]. For colloidal nanostructured SERS substrates, the reproducibility of the SERS signal is a concern due to the possible aggregation of nanoparticles [47]. Solid-based nanostructured substrates composed of regular nanostructures can improve the reproducibility of SERS detection. The fabrication strategy of solid-based substrates included chemical self-assembly [63], the in situ growth of nanostructures [64], electrochemical deposition [65] and magnetron sputtering [66]. Many studies have proven that the enhancement effect of SERS substrates is strongly influenced by the type of materials, the structures’ size, shape and surface morphology, and the interaction modes between detection samples and SERS substrates. A variety of materials, such as gold [67], silver [68], copper nanoparticles [69], metal nanocomposites [70], core–shell nanomaterials [71], carbon materials [8], metal–organic frameworks [72] and magnetic nanomaterials [73], have been used to prepare SERS substrates. Compared to nanoparticles with a single element, metal composite nanostructures can greatly improve SERS’s enhancement performance, and bimetallic core–shell nanostructures have the function of regulating localized surface plasmon resonance (LSPR) characteristics through the alterations of elements and configuration [74,75]. For example, Krishnan et al. [76] synthesized bimetallic Ag@Au nanodisks, which showed greatly enhanced SERS performance with an enhancement factor up to 0.47 × 10^5^ (Figure 2A). Zhou et al. [77] reported smart triple-functional Au–Ag-stuffed nanopancakes (AAS-NPs) with multiple functions for bacteria sensing, inactivation in human blood samples and bacterial biofilm disruption (Figure 2B). SERS substrates made of magnetic nanocomposites can be used to separate and enrich target analytes in complex samples with the help of an external magnetic field, which not only achieves the pretreatment of complex matrix samples but also improves the SERS detection sensitivity of target analytes. Wang et al. [78] reported a novel strategy for the synthesis of magnetic-based flower-like silver composite microspheres (Fe_3_O_4_@SiO_2_@Ag microflowers) with good dispersion, good magnetic responsiveness and high reproducibility. The aptamer-functionalized microflowers were applied for the capture of *Staphylococcus aureus* in tap water and significantly enhanced the SERS signal (Figure 2C). Graphene is an excellent alternative for the fabrication of SERS substrates due to its two-dimensional flat structure, uniform electronic and photonic properties, excellent mechanical stability, atomic uniformity and good biocompatibility [79]. In addition, graphene nanocomposites can improve the adsorption efficiency of target analytes and also reduce fluorescence background interference due to their large surface area and fluorescence quenching abilities. Meng et al. [80] developed a kind of high-performance SERS substrate made of graphene (G)–silver nanoparticle (AgNP)–silicon (Si) sandwich nanohybrids (G@AgNPs@Si) (Figure 2D). Since AgNPs were regularly arranged on the graphene surface, this kind of SERS substrate had good stability and reproducibility and was not only capable of adenosine triphosphate (ATP) molecular detection with a detection limit of 1 pM but also achieved the capture, discrimination and inactivation of bacteria. By making full use of electromagnetic enhancement and chemical enhancement mechanisms, the design and optimization of substrate nanostructures are an effective approach to achieving highly efficient bacteria detection through SERS.

## 4. The SERS Strategy for Bacteria Detection

SERS is an ideal bacteria detection method with the characteristics of high efficiency, fast analysis speed and high sensitivity. A SERS signal can reveal rich fingerprint information about bacterial compositions including nucleic acids, proteins, lipids and pigments [81]. The SERS-based methods for bacteria detection are usually divided into two strategies: the label-based method and the label-free method [82]. Label-based detection methods usually use the SERS tag as a quantitative reporter for the ultrasensitive detection of bacteria [83]. While label-free SERS detection is easy to operate due to the fact that it has no requirements for specific detection labels [84]. Table 1 summarized the detailed information concerning the bacteria detection via SERS, including detection strategy, SERS substrate type, detection limit and range, excitation wave-length, characteristic peak and practicability.

### 4.1. The Label-Free SERS for Bacteria Detection

**Figure 3:**
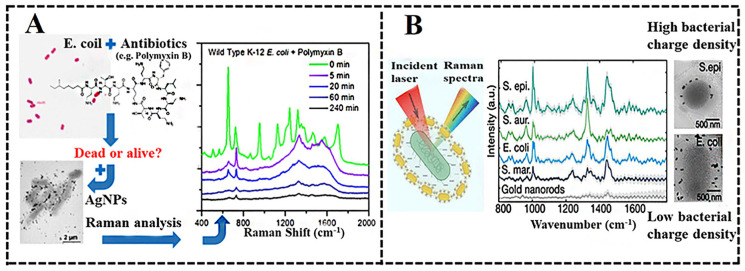
(**A**) Rapid detection and discrimination between live and dead bacteria via label−free SERS after coating AgNPs [85]. Reproduced with permission from American Chemical Society Copyright (2022); (**B**) SERS detection of four different bacteria using gold nanorod particles [87]. Reproduced with permission from American Chemical Society Copyright (2022).

In the case of the label-free method, the bacteria samples are directly mixed with the SERS-active nanoparticle solution or are placed in contact with the surface of the solid nanostructure SERS substrate, so as to enhance the Raman signal of the bacteria. The characteristic of label-free SERS is that it directly identifies bacteria according to the intrinsic vibrational fingerprints of pathogenic bacteria. The SERS signal of bacteria in the label-free method is easily interfered with by a complex matrix. Ag or Au nanoparticle-based SERS substrates are usually applied for the label-free detection of bacteria strains. For example, Zhou et al. [85] used Ag nanoparticles as SERS substrates to rapidly detect SERS signals of *Escherichia coli* at the excitation wavelength of 633 nm and successfully discriminate between live and dead bacteria by using SERS mapping. The wild-type strains and antibiotic-resistant bacterial strains of *Escherichia coli* were also successfully distinguished by the SERS method (Figure 3A). In another study, Zhou et al. [86] employed the synthesized silver nanoparticles to discriminate three strains of *Escherichia coli* and one strain of *Staphylococcus epidermidis* by hierarchy cluster analysis. These studies opened an avenue for developing SERS-based strategies for bacteria detection and discrimination. Tadesse et al. [87] synthesized gold nanorods with five different sizes and demonstrated consistent bacteria detection in liquid using SERS with large-area enhancement. The plasmonic interactions between gold nanorods and four different bacteria were investigated by varying the concentration ratios of bacteria and nanorods. Their research revealed that the surface charge of the bacteria membrane affected nanoparticle binding affinities, thereby affecting the SERS signal enhancement of bacteria (Figure 3B). The discrimination of bacteria species by some characteristic peaks in SERS spectra was full of challenges owing to the extremely similar chemical components of different bacteria. Aptamer, which is essentially a group of single-strand DNA, was coated on SERS-active nanoparticles and bound to the target bacteria specifically through non-covalent bonds. Specific SERS spectra for target bacteria can be obtained by the recognition of aptamer and bacteria. At present, aptamers for the specific binding of various bacteria have been screened [88]. By bonding bacteria aptamers with SERS-active nanoparticles, highly sensitive and specific detection of target bacteria can be achieved. Gao et al. [89] developed a quick and direct method for the recognition and detection of bacteria through SERS based on bacteria-aptamer@AgNPs. The SERS signal of *Staphylococcus aureus* was dramatically enhanced by its specifically recognized aptamer, and the detection limit was down to 1.5 CFU/mL. The linear relationship between the bacterial concentration and the peak intensity at 735 cm^−1^ was obtained, and the linearity range was from 10 to 10^7^ CFU/mL. Recently, magnetic SERS substrates have received extensive attention due to their dual functions of concentrating target bacteria in solution samples under an external magnetic field and enhancing bacteria’s SERS signals based on the localized electromagnetic enhancement of metallic nanoparticles. Wang’s group reported a capture–enrichment–enhancement (CEE) three-step method for label-free SERS detection of different bacteria with a detection limit of 10^3^ CFU/mL for *Escherichia coli* and *Staphylococcus aureus* according to the strongest Raman peak at 729 cm^−1^ [90]. They fully utilized the capture and enrichment ability of polyethylenimine-modified, Au-coated magnetic microspheres (Fe_3_O_4_@Au@PEI) and synergistically SERS’s enhancement capability of Fe_3_O_4_@Au@PEI microspheres and Au@AgNPs. Their reported SERS method could complete bacteria detection within 10 min and had great application potential in detecting bacteria pathogens in complex solutions. The above-mentioned SERS substrates for bacteria detection are mostly zero-dimensional nanoparticles or one-dimensional nanorods, which are easy to agglomerate, thus affecting the reproducibility of bacteria detection through SERS.

**Figure 4:**
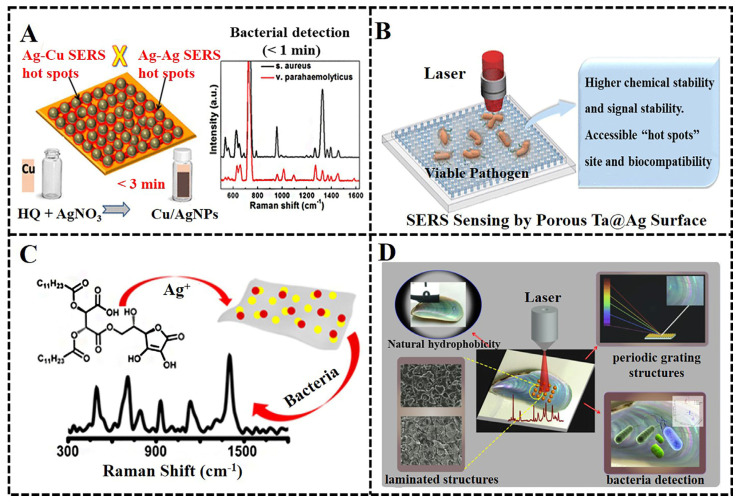
(**A**) Schematic illustration of the fabrication of Cu/Ag nanoparticle substrate and its application for label-free SERS detection of bacteria [93]. Reproduced with permission from American Chemical Society Copyright (2022); (**B**) Schematic illustration of solid-state bimetallic Ta@Ag substrate for efficient SERS sensing of *Escherichia coli* [94]. Reproduced with permission from American Chemical Society Copyright (2022); (**C**) Schematic illustration of effective SERS detection of *Pseudomonas aeruginosa* via SERS-active flexible polydimethylsiloxane (PDMS) films [95]. Reproduced with permission from American Chemical Society Copyright (2022); (**D**) Schematic illustration of a natural surface SERS substrate based on natural mussel shells for the detection and discrimination of pathogenic bacteria [96]. Reproduced with permission from American Chemical Society Copyright (2022).

To improve the reproducibility and reliability of SERS detection for pathogenic bacteria, it is necessary to design high-density SERS hot spots based on 2D planar SERS substrates and 3D plasmonic nanostructures [91]. Two-dimensional SERS substrates are usually prepared by depositing a uniform layer of SERS-active nanoparticles on the surface of supporting substrates such as glass, paper or polymer under controllable conditions [92]. Three-dimensional plasma nanostructures, such as nanopillar or nanotube arrays, are SERS-active structures in three-dimensional space, that can be fabricated with precise geometric control and positional arrangement of plasmonic nanoparticles or other nanoscale materials. Silver-based nanostructures have been widely used to prepare SERS substrates owing to their high plasmonic activity, relatively low cost among noble metals and ideal dielectric constant. For example, Beyene et al. [93] used Cu-foil, silver nitrate and hydroquinone to successfully build a substrate with Cu/Ag nanoparticles in a reusable and cost-effective method. Their fabricated substrate for label-free SERS bacteria detection showed a high detection sensitivity owing to the second and third-generation SERS hot spots through the cooperative interaction of homogeneous (Ag–Ag) and heterogeneous (Ag–Cu) surfaces (Figure 4A). The strongest peak at 730 cm^−1^ can be detected when the *Staphylococcus aureus* and *Vibrio parahaemolyticus* concentrations are as low as 10^4^ CFU/mL. Chen et al. [94] constructed a solid-state bimetallic film of Ta@Ag with a porous structure via magnetron sputtering technology for *Escherichia coli* detection with a detection limit of 100 CFU/mL. Bimetallic Ta@Ag SERS substrate improved the stability and biocompatibility for efficient SERS sensing of bacteria (Figure 4B). A SERS-active flexible polydimethylsiloxane (PDMS) film containing carbon dots and silver nanoparticles was fabricated by Jelinek’s group, which showed a remarkable SERS signal compared to conventional SERS probes. The detection of *Pseudomonas aeruginosa* was demonstrated with a limit of 10^4^ CFU/mL (Figure 4C) [95]. Inherent homogeneous three-dimensional nanostructures of natural materials, such as taro leaf, mussel shell and cicada wings, have been applied in the preparation of SERS substrates. A highly efficient and reproducible SERS substrate could be constructed by assembling metallic nanoparticles into the 3D nanostructures of natural materials. Yuan et al. [96] developed a natural surface SERS substrate by the self-assembly of size-tunable Au@AgNPs on the surface of natural mussel shells. The mussel shell SERS substrate showed a high electromagnetic field enhancement effect and had good reproducibility, which made it successfully applicable for the discrimination of different kinds of pathogenic bacteria (*Escherichia coli*, *Pseudomonas aeruginosa* and *Staphylococcus aureus*) (Figure 4D). For label-free SERS bacteria detection, a sensitive and stable SERS substrate was essential to capture reproducible SERS bacteria signals for the detection and identification of bacteria species.

### 4.2. The Use of SERS Tags for Bacteria Detection

**Figure 5:**
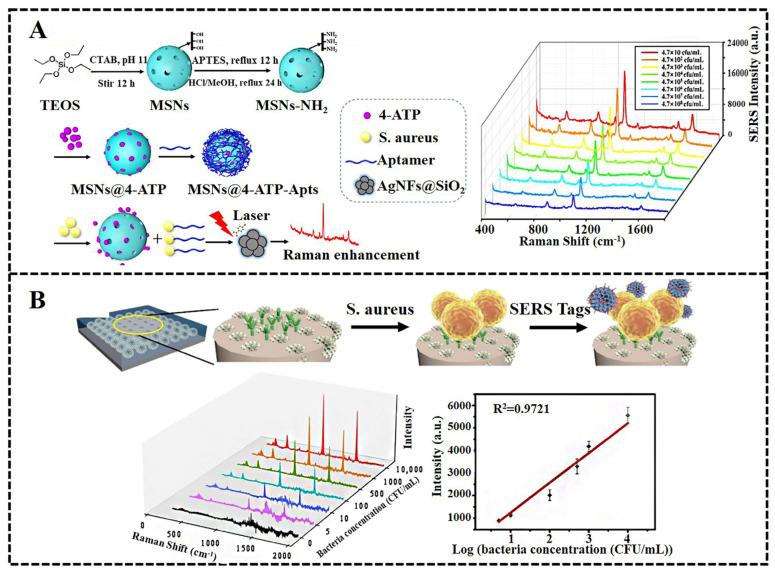
(**A**) Schematic illustration of *Staphylococcus aureus* detection based on the target−responsive release of 4−ATP molecules from aptamer−gated MSNs and the collected SERS spectra of *Staphylococcus aureus* [99]. Reproduced with permission from American Chemical Society Copyright (2022); (**B**) Schematic illustration of *Staphylococcus aureus* sensing based on the liquid−infused, patterned nanocoating using SERS tag and the linear response between Raman intensity of reporter signal and the *Staphylococcus aureus* concentration [100]. Reproduced with permission from American Chemical Society Copyright (2022).

Multiple peaks appeared when measuring the Raman signal of one type of bacterium, as can be found in the previous discussions. Bacteria detection becomes more complicated when there are a variety of bacteria in complex environments and the distinguishment of bacteria from complex samples is a huge challenge. Therefore, the selective and quantitative detection of pathogenic bacteria is difficult to achieve with the label-free SERS detection method. To solve this problem, various SERS tags for the label-based SERS detection of bacteria have been developed. A SERS tag is commonly composed of SERS-active nanoparticles (AgNPs or AuNPs) and Raman reporter molecules. In SERS tags, SERS-active nanoparticles are usually functionalized with molecules (antibody, aptamer, vancomycin, etc.) for specific recognition and direct binding of target bacteria. Highly sensitive quantitative detection of target bacteria can be realized owing to the strong and stable SERS signal of Raman reporter molecules. Bi et al. [97] developed a SERS-active Au@Ag core–shell nanorod (Au@AgNR) tag for the quantitative detection of *Escherichia coli*. According to the change in peak intensity at 1517 cm^−1^, the detection limit for *Escherichia coli* was as low as 100 CFU/mL, with a detection range of 10^2^–10^7^ CFU/mL. These results demonstrated that the SERS tag-based analysis platform possessed good reproducibility and high specificity, and it had a high binding affinity to *Escherichia coli*. Furthermore, the authors applied the SERS nanotag-based detection platform to successfully discriminate antibiotic resistance with the help of PCA chemometric analysis. In order to further improve the capture efficiency of target bacteria, magnetic nanoparticles (MNPs) were employed to prepare SERS tags. Zhang et al. [98] reported an aptamer-functionalized SERS tag (vancomycin-modified Fe_3_O_4_@Au magnetic nanoparticles) for the rapid capture and simultaneous detection of two pathogenic bacteria in authentic specimens. On the basis of dual recognition by vancomycin and aptamers, the capture efficiency was as high as 88.89% for *Staphylococcus aureus* and 74.96% for *Escherichia coli*. By detecting the characteristic peak intensities (*Staphylococcus aureus* at 1074 cm^−1^ and *Escherichia coli* at 1331 cm^−1^) of the SERS tags, the linear calibration curves for *Staphylococcus aureus* and *Escherichia coli* were obtained, with a limit of detection of 20 and 50 CFU/mL, respectively. Due to its large surface area and high-density pores, mesoporous silica is an ideal candidate for coating more bacteria-recognizing aptamers to improve SERS sensitivity. Zhu et al. [99] reported a novel and sensitive approach for the quantitative detection of *Staphylococcus aureus* using SERS technology based on the target-induced release of signal molecules from aptamer-gated, aminated, mesoporous silica nanoparticles. A linear relation between the peak intensity of 4-ATP at 1071 cm^−1^ and the concentration of *Staphylococcus aureus* was obtained in the range of 4.7 × 10 to 4.7 × 10^8^ CFU/mL, and the detection limit was as low as 17 CFU/mL (Figure 5A). Wang et al. [100] synthesized a DPSNs-Au-MBA-aptamer as a SERS tag for the recognition of *Staphylococcus aureus* by modifying the Raman reporter 4-mercaptobenzoic acid (4-MBA) and the aptamer on the surface of dendritic porous silica nanoparticles (DPSNs). They prepared a slippery, patterned, liquid-infused nanocoating on the glass substrate. The combination of the liquid-infused patterned nanocoating with the DPSNs-Au-MBA-aptamer SERS tag achieved highly sensitive SERS detection of *Staphylococcus aureus* with a low detection limit of 2.6 CFU/mL. A linear response relationship between bacterial concentrations ranging from 5 to 10^4^ CFU/mL and Raman intensity at the peak of 1079 cm^−1^ was also obtained (Figure 5B). SERS tags have also attracted great attention in their application for bacteria imaging and phototherapies in the past few years. Gao et al. [101] developed a multifunctional SERS platform composed of a gold film SERS-active substrate and the SERS tag of vancomycin-modified core−shell Prussian blue-coated gold nanoparticles. The developed SERS tag-based technique could quantitatively detect *Staphylococcus aureus* with a wide detection range of 10 to 10^7^ CFU/ mL. A multifunctional aldehyde group that conjugated Au@Rubpy /GO SERS tags was fabricated by Lin et al. [102] for the optical labeling and photothermal ablation of bacteria. This SERS tag could realize sensitive Raman imaging of *Staphylococcus aureus* and *Escherichia coli* and indicated the possibility of measuring antibacterial response during the photothermal process.

Compared with label-free SERS, the label-based detection method has relatively high sensitivity and selectivity due to the dual functions of SERS tags, including the capture of the target bacteria and enhancement of the Raman signal. Therefore, label-based SERS detection is usually applied in the quantitative analysis of target bacteria in complex biological samples. The disadvantages of label-based detection are that the preparation of SERS tags is complex and time-consuming. On the contrary, label-free SERS detection has great advantages in distinguishing bacterium types according to the differences in the SERS spectrum, and it is unlikely to be used for the accurate quantitative detection of bacteria. Unlike small molecules, bacterial cells are micron-sized samples, and they are usually inhomogeneous, thus bringing several difficulties to the SERS detection of bacteria. Currently, the problems to be solved in bacteria detection through SERS can be summarized as follows: (1) The detection limit of bacteria using SERS is usually high. Therefore, new strategies for effectively enriching and focusing the laser on bacteria are urgently needed in order to eliminate background interference and reduce the SERS detection limit. (2) The poor quantitative ability of bacteria detection through SERS is another challenge due to the limited range of light spots of Raman spectrometers. SERS mapping as a semi-automatic data collection technique can be applied to improve the accuracy of quantitative bacteria detection by automatically collecting hundreds of spectra at every pixel in a wide detection area. Based on SERS mapping techniques and SERS tags, the imprinted SERS mapping platform developed by Yang et al. [103] showed good quantitative analysis abilities for bacteria with a wide linear range of 10^2^ to 10^8^ CFU/mL and a low detection limit of ~1.35 CFU/mL for Escherichia coli. (3) The distinguishment of the similar spectra from different bacteria. A potential solution to this bottleneck is an algorithmic analysis technique, such as principal component analysis (PCA), principal component regression (PCR), multiple linear regression (MLR) or linear discriminant analysis (LDA). In addition, artificial intelligence (AI) and machine learning (ML) are receiving increasing attention in the application of the processing of SERS bacteria data [104] to realize automatic data processing. There is still a long way to go to address all these issues in the SERS detection of bacteria so that SERS can truly be used as a reliable and robust tool for clinical diagnosis and environmental bacterial pollution monitoring.

**Table 1:** Summary of representative bacteria detection via SERS.

Category	SERS Substrate	Targets	LOD	Excitation Wavelength	Characteristic Peak	Detection Range	Sample	Practicability	Reference
label-free SERS detection	Ag nanoparticles	*Escherichia coli*	-	633 nm		-	culture solutions	live or dead bacteria discrimination	[85]
	Ag nanoparticles	*Escherichia coli* and *Staphylococcus epidermidis*	-	633 nm		-	culture solutions	bacteria discrimination	[86]
	gold nanorods	*Escherichia coli* and *Staphylococcus aureus*	-	785 nm		-	water	bacteria identification	[87]
	aptamer@AgNPs	*Staphylococcus aureus*	1.5 CFU/mL	632.8 nm	735 cm^−1^	10 to 10^7^ CFU/mL	culture solutions	in situ bacteria detection	[89]
	Fe_3_O_4_@Au@PEI	*Escherichia coli* and *Staphylococcus aureus*	10^3^ CFU/mL	785 nm	729 cm^−1^	-	PBS solution	-	[90]
	Cu/Ag nanoparticles solid substrate	*Staphylococcus aureus* and *Vibrio parahaemolyticus*	10^4^ CFU/mL	785 nm	730 cm^−1^		culture solutions	-	[93]
	Ta@Ag bimetallic film	*Escherichia coli*	10^2^ CFU/mL	633 nm	1396 cm^−1^		culture solutions	-	[94]
	hybrid C-dot-Ag films	*Pseudomonas aeruginosa*	10^4^ CFU/mL	633 nm	1400 cm^−1^ and 930 cm^−1^	-	culture solutions	-	[95]
	Au@Ag nanoparticles on mussel shell	*Escherichia coli*, *Staphylococcus aureus* and *Pseudomonas aeruginosa*	-	633 nm	-	-	culture solutions	discrimination bacteria	[96]
detection based on SERS tags	Au@Ag core–shell nanorod	*Escherichia coli*	10^2^ CFU/mL	785 nm	1517 cm^−1^	10^2^ to 10^7^ CFU/mL	PBS solution	antibiotic susceptibility testing	[97]
	Fe_3_O_4_@Au-Van MNPs	*Staphylococcus aureus* and *Escherichia coli*	20 and 50 CFU/mL	785 nm	1074 cm^−1^ and 1331 cm^−1^	20 to 10^5^ CFU/mL and 50 to 10^5^ CFU/mL	PBS solution	-	[98]
	aptamer-gated, mesoporous silica nanoparticles	*Staphylococcus aureus*	17 CFU/mL	785 nm	1071 cm^−1^	4.7 × 10 to 4.7 × 10^8^ CFU/mL	fish meat	-	[99]
	DPSNs-Au-MBA-aptamer	*Staphylococcus aureus*	2.6 CFU/mL	785 nm	1079 cm^−1^	5 to 10^4^ CFU/mL	PBS solution	bacteria repellence and sensing	[100]
	silver nanoparticles	pathogenic *mycobacteria*	-	532 nm	-	-	TE buffer	bacteria discrimination	[105]
microfluidic SERS	silver film	*Escherichia coli*	10^3^ CFU/mL	632.8 nm	740 cm^−1^	10^3^ to 10^6^ CFU/mL	culture solutions	bacteria capture	[106]
	AgNPs	*Escherichia coli* and *Staphylococcus aureus*	3 and 3.5 CFU/mL	532 nm	1362 cm^−1^ and 1613 cm^−1^	10 to 10^7^ CFU/mL	whole blood	bacteria infections diagnosis	[107]

## 5. The Microfluidic SERS Chip for Bacteria Detection and Antimicrobial Susceptibility Testing

Over the past decades, the microfluidic chip, which is also called “lab on a chip”, has attracted extensive attention in the fields of food safety [108], environmental monitoring [109] and disease diagnosis [110]. Microfluidic chips, as a potential analysis platform, can offer lots of benefits, such as high throughput, low sample consumption, in situ monitoring and multifunctional integration [111,112]. In addition, microfluidic chip systems allow the precise manipulation of small volumes of fluids, which is used to overcome the low consistency and repeatability of SERS-based detection in unstable experimental conditions [113,114]. Microfluidic SERS chips are fabricated by integrating SERS-active nanostructures into microfluidic devices [115,116]. By making full use of the advantages of microfluidic chips and SERS, microfluidic SERS chips can not only provide highly reproducible SERS spectra for bacteria detection but also integrate different functional units for the separation and enrichment of target bacteria in complex fluid samples [117]. In recent years, with the rapid development of micro/nanofabrication technology, microfluidic SERS chips have shown great potential for the detection and identification of pathogenic bacteria and antimicrobial susceptibility testing (AST) [118].

### 5.1. The Microfluidic SERS Chip for Bacteria Detection

The key purpose of microfluidic SERS chips is the integration of SERS-active nanostructures in microfluidic channels. The integration strategy of SERS-active nanostructures in microfluidic chips can be divided into two categories: (1) the droplet-based strategy, by mixing the sample solution with colloidal nanoparticles in microdroplets; (2) the substrate strategy, by constructing solid nanostructures on the microchannel substrate as the Raman enhancement substrate. Some studies have shown that laser processing can be applied to build solid SERS substrates in the microchannel. For example, Bai et al. [119] determined that a layered Cu–Ag nanodot array could be fabricated in microfluidic channels by using all-femtosecond laser processing. This SESR substrate exhibited a high enhancement factor of 7.3 × 10^8^ and could detect Cd^2+^ ions at concentrations as low as 10 ppb. Femtosecond laser direct writing (FsLDW) has the capability to fabricate and integrate metal or alloy nanostructures in the microfluidic chip. An Ag/Pd alloy nanostructure with a high enhancement factor of about 2.62 × 10^8^ was integrated into the microfluidic chip by Ma et al. [120] using the FsLDW technology.

**Figure 6:**
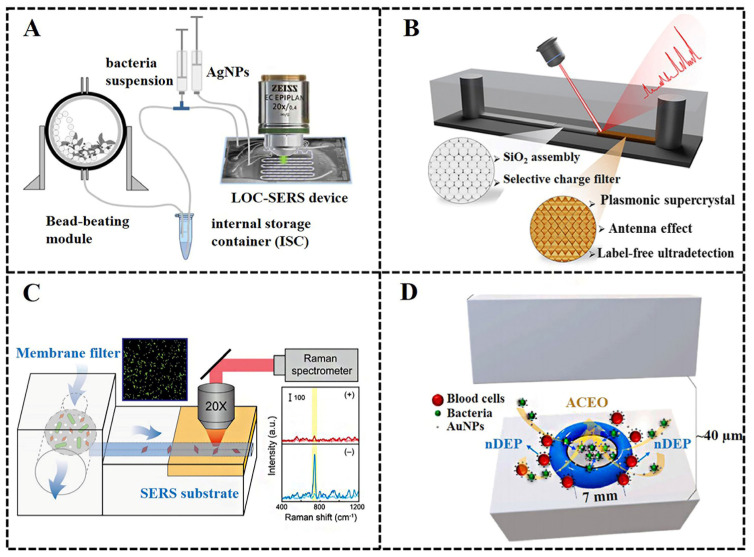
(**A**) Schematic illustration of droplet-based microfluidic device for bacteria detection including the sample lysing module (bead-beating system) and SERS detection module based on AgNPs [105]. Reproduced with permission from American Chemical Society Copyright (2022); (**B**) Schematic representation of the microchip integrated with solid plasmonic super-crystal by injecting colloidal dispersion gold nano-octahedrons in microchannel [121]. Reproduced with permission from American Chemical Society Copyright (2022); (**C**) Schematic diagram of the microfluidic system integrated with membrane filtration and SERS-active substrate [106]. Reproduced with permission from American Chemical Society Copyright (2022); (**D**) Schematic diagram of multi-functional microfluidic system integrated with three-dimensional alternative current electrokinetic and SERS detection for bacteria sensing [107]. Reproduced with permission from Elsevier B.V. Copyright (2022).

Droplet-based microfluidic chips provide a good platform for mixing analytes with colloidal nanoparticles. Mühlig et al. [105] designed a closed droplet-based lab-on-a-chip (LOC) device to mix silver nanoparticles with bacteria suspension in droplets. An automated, closed system for the mixing and SERS measurement of bacterial samples was designed by combining the sample lysing module with a microfluidic chip device, which allowed the efficient collection of SERS spectra of pathogenic mycobacteria. With the aid of their SERS data sets and principal component analysis (PCA), six kinds of pathogenic mycobacteria were successfully distinguished by the hierarchical model with a high accuracy of 93% (Figure 6A). When colloid-based nanoparticles are employed as SERS substrates, the ratio of colloidal nanoparticles to the samples and the mixing efficiency are often influenced by the flow rates, which bring about potential instability between droplets. In addition, movement and coalescence of droplets will happen occasionally, causing difficulties in SERS measurements.

These limitations can be resolved by constructing solid-state SERS-active substrates in microchannels by chemical self-assembly or vapor deposition technology. Yan et al. [115] developed a one-step electroless galvanic replacement reaction to synthesize Ag nanostructure SERS substrates in a microfluidic channel for the label-free sensing of chemical molecules and biomolecules. García-Lojo et al. [121] designed a SERS-based microfluidic chip for the direct analysis of pyocyanin secreted by *Pseudomonas aeruginosa*. In the microfluidic channel, a solid 3D plasmonic super-crystal with high “hot-spots” density was formed by the microfluidic-induced assembly of gold nano-octahedrons. The SERS-based microfluidic chip exhibited outstanding sensing performance, with a detection limit as low as 10^−9^ M for pyocyanin (Figure 6B). Filter membranes and label-free SERS sensing can be easily integrated with microfluidic chips for the enrichment or encapsulation of bacterial cells. Chang et al. [106] developed a microfluidic system integrated with membrane filtration and a SERS-active substrate for *Escherichia coli* enrichment, metabolite collection and in situ SERS measurements. The SERS-active substrate integrated into the chip was fabricated by silver evaporation technology on a glass slide. The large variation in the SERS signal was overcome by the membrane filtration and SERS (MF-SERS) system. The detection limit of the MF-SERS system for bacteria was 10^3^ CFU/mL, and the detection range decreased to 10^3^ CFU/mL from 10^6^ CFU/mL (Figure 6C). Based on the interactions between dielectric particles and the electric field, the dielectrophoresis (DEP) technique can manipulate microscale bioparticles, including the separation, sorting and trapping of bacterial cells [122]. DEP has been widely used in microfluidic systems to separate and concentrate rare bacterial cells [123,124]. Chen et al. [107] reported a microfluidic chip combining DEP enrichment and SERS detection for bacteria separation, identification and antibiotic susceptibility testing in a rapid and simple fashion. The microfluidic SERS chip exhibited high sensitivity and was able to discriminate different bacteria in whole blood by integrating AC electrokinetics into the chip. It showed a detection limit of 3 CFU/mL for *Staphylococcus aureus* and 3.5 CFU/mL for *Escherichia coli*, and the detection range for both was from 10 to 10^7^ CFU/mL (Figure 6D). While possessing unique merits, such as high throughput measurements, low sample consumption, multifunctional integration and increased repeatability, microfluidic SERS also brings challenges, including difficulty in chip sealing or in situ fabrication and extra set-up required for controlling the fluid delivery.

### 5.2. The Microfluidic SERS Chip for Antimicrobial Susceptibility Testing

Although antibiotics can effectively treat infections from pathogenic bacteria, drug-resistant bacteria are emerging as a critical global issue due to the abuse of antibiotics. Antimicrobial susceptibility testing (AST) is a standard laboratory procedure for the assessment of the antibiotic resistance of bacteria to avoid the emergence of drug-resistant strains [125]. Conventional AST methods, such as the broth dilution method and Kirby–Bauer disk diffusion tests, usually require time-consuming sample preparation steps. The clinical treatment is severely delayed in this case [126]. Therefore, rapid and sensitive methods for AST are of great significance for clinical antibiotic therapy. The SERS technique is considered to be a rapid and sensitive tool to monitor bacterial response to antibiotic treatment by detecting the metabolism of bacteria as an alternation [127]. Considering the advantages of microfluidic chips, the use of microfluidic SERS chips for AST is not only time-saving and sensitive but also allows for high-throughput detection.

**Figure 7:**
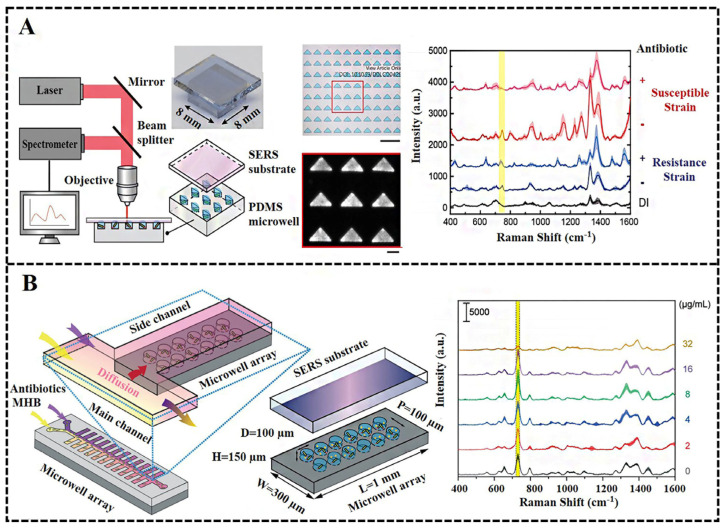
(**A**) Schematic diagram of microwell–SERS system for rapid and high-throughput AST of susceptible and resistant *Escherichia coli* treated with and without kanamycin at 16 µg/mL [131]. Reproduced with permission from Royal Society of Chemistry Copyright (2022); (**B**) Schematic of the antibiotic concentration gradient device (the blue-framed inset shows the detailed microwell array and antibiotic concentration layout) and the average SERS spectra for ampicillin-susceptible bacteria treated with ampicillin at different concentrations [131]. Reproduced with permission from Elsevier B.V. Copyright (2022).

A microfluidic device integrated with SERS technology (the microwell–SERS system) was developed by Huang et al. [128] for rapid and high-throughput AST (Figure 7A). Highly dense (100 wells/mm^2^) and independent triangular microwells were fabricated using polydimethylsiloxane (PDMS) for multi-parallel SERS measurements. Bacterial cells, after treatment with antibiotics, could be confined and enriched in the microwells, and the AST time was shortened greatly due to the high sensitivity of SERS for the detection of their metabolisms. The bacterial concentration was reflected by a peak intensity of 740 cm^−1^; a characteristic peak attributed to bacteria-secreted metabolites, adenine and hypoxanthine. High-throughput and in situ SERS measurements were accomplished by attaching a silver-island film as a SERS-active substrate on the top of the microwells. The antibiotic susceptibility of *Escherichia coli* and *Staphylococcus aureus* was determined by the difference in I_740_ before and after the antibiotic treatment. In the operation process of AST, the preparation of antibiotics with a series of concentrations is an important step in investigating bacteria responses for the determination of the minimum inhibitory concentrations (MIC). A drawback of the proposed microwell–SERS system is the requirement of the centrifugal operation to disperse bacteria and antibiotics inside the microwells. To overcome this problem, a concentration gradient microfluidic device composed of a Y-shaped main channel with 66 side channels and a microwell array was reported by Lin et al. [129] for high-throughput AST through SERS (Figure 7B). The Y-shaped main channel and a series of side channels were designed to generate a concentration gradient of antibiotics by mixing a high-concentration antibiotic solution with a pure culture medium based on laminar flow and diffusion behavior. A microwell array placed under the side channels was used for bacteria introduction, medium washing and the isolation of microwells. The SERS measurement of bacteria was performed by placing a uniform silver film SERS substrate on the bottom of the microwells. All AST operation steps, including bacteria loading, antibiotic concentration generation, buffer washing and isolated bacteria growth in microwells with different antibiotic concentrations, were performed in this chip. The SERS-based AST assay using the concentration gradient generation device only required 20 μL of bacteria solution and 5 h of operation time and overcame the tedious and labor-intensive procedures in conventional AST methods. The efficiency of SERS-based AST was further improved with an automated microfluidic control system [130]. All AST procedures, including antibiotic treatment, bacteria culture and isolation, as well as high-throughput SERS measurements, were integrated into the single microfluidic device. The operating process of the SERS–AST protocol was divided into seven steps comprising four parts: antibiotic preloading, bacteria injection for antibiotic reconstitution and incubation, DI water washing and air isolation and SERS measurement. SERS signal interference from the culture medium was minimized since bacteria were localized in the microwell. For the automatic AST–SERS microfluidic system, the entire SERS–AST protocol from bacteria injection to final SERS measurement only required 3.5 h, which is much faster than traditional culture-based methods. This method had the ability to discriminate ampicillin-susceptible and -resistant *Escherichia coli* strains by the difference in I_733_ signals.

## 6. Conclusions and Future Perspectives

Rapid and accurate bacteria detection is essential for food quality evaluations, environmental quality assessments and clinical antibacterial treatments. Herein, we reviewed the development and applications of SERS as a powerful tool for the rapid and sensitive detection of various bacteria. The key to SERS analysis of bacteria is to prepare an ideal SERS substrate with a high enhancement factor and good signal reproducibility, as well as a low cost. Taking advantage of electromagnetic and chemical enhancement mechanisms, the design of SERS-active nanostructures with a high density of “hot-spots” is worth continuously developing for higher sensitivity and reliability in the SERS detection of bacteria. The compositions of proteins, lipids and polysaccharide molecules for different bacteria species present different SERS fingerprint spectra. Therefore, the label-free SERS method is undoubtedly the most ideal way to identify bacterial species. However, the discrimination of Raman spectra in complex samples is a challenge. In recent years, various SERS tags composed of the Raman reporter and recognition element have been developed. Label-based SERS methods are often used for the capture and highly sensitive detection of target bacteria in complex real-world samples such as blood, urine and sewage. However, the operation processes of label-based SERS methods are usually complicated, and the physiological state of bacterial cells may also be affected due to the addition of SERS probes. The challenge of label-based SERS for bacteria detection is how to simplify the synthesis and operation processes of SERS tags, so as to expand their practical applications in the future. It is inevitable to develop the accurate quantitative analysis of bacteria samples for both label-free and label-based SERS methods since the concentration or density of a certain type of bacterium is needed.

The microfluidic chip, as a powerful miniaturized device, possesses many advantages, including high throughput, low sample consumption and a controllable microenvironment. The combination of SERS with microfluidic chips provides an effective method for bacterial analysis with the multi-functions of separation, enrichment and highly sensitive detection. Various types of microfluidic SERS chips for bacterial enrichment and detection were summarized in this review. The emergence of antibiotic resistance is a great threat to global health. AST is a standardized method to guide the rational use of antibiotics so as to slow down the emergence of resistant strains. Microfluidic SERS chips integrated with a microwell array or concentration gradient generator are able to achieve high-throughput AST detection and shorten the culture time of bacteria. Despite the progress in the SERS-based microfluidic chips for bacteria detection and AST, there are still some challenges, such as the interference from the complex matrix, the limitations in practical applications and the requirement of an external Raman spectrometer. On the other hand, the lack of standard Raman databases of bacteria makes it difficult to obtain correct analysis and discrimination. In the future, more efforts should be taken to develop automated, SERS-based, microdevice analysis platforms and machine learning for real-world applications in clinical diagnostics of bacterial infection-related diseases.

## Data Availability

The data presented in this study are available on request from the corresponding author.
